# Adenovirus associated with acute diarrhea: a case-control study

**DOI:** 10.1186/s12879-018-3340-1

**Published:** 2018-09-03

**Authors:** Fang-zhou Qiu, Xin-xin Shen, Gui-xia Li, Li Zhao, Chen Chen, Su-xia Duan, Jing-yun Guo, Meng-chuan Zhao, Teng-fei Yan, Ju-Ju Qi, Le Wang, Zhi-shan Feng, Xue-jun Ma

**Affiliations:** 1 Hebei Medical University, Shijiazhuang, 050031 Hebei China; 2 Key Laboratory for Medical Virology, National Health and Family Planning Commission, National Institute for Viral Disease Control and Prevention, Chinese Center for Disease Control and Prevention, No. 155 Changbai Street, Chang ping District, Beijing, 102206 China; 3 Children’s Hospital of Hebei Province, Shijiazhuang, 050031 Hebei China

**Keywords:** Diarrhea, HAdV, Case-control, Serotypes

## Abstract

**Background:**

Diarrhea is a major source of morbidity and mortality among young children in low-income and middle-income countries. Human adenoviruses (HAdV), particular HAdV species F (40, 41) has been recognized as important causal pathogens, however limited data exist on molecular epidemiology of other HAdV associated with acute gastroenteritis.

**Methods:**

In the present preliminary study, we performed a case-control study involving 273 children who presented diarrheal disease and 361 healthy children matched control in Children’s hospital of Hebei Province (China) to investigate the relationship between non-enteric HAdV and diarrhea. HAdV were detected and quantified using quantitative real-time PCR (qPCR) and serotyped by sequencing and phylogenetic analysis. Odds ratio (OR) was used to assess the risk factor of HAdV.

**Results:**

HAdV were detected in 79 (28.94%) of 273 children with diarrhea including 7 different serotypes (HAdV 40, 41, 3, 2,1,5 and 57) with serotypes 40, 41 and 3 being the most dominant and in 26 (7.20%) of 361 healthy children containing 9 serotypes (HAdV 40, 41, 3, 2,1,5,57,6 and 31). A majority (91.14%) of HAdV positives occurred in diarrhea children and 65.38% in controls< 3 years of age. No significant difference in the viral load was found between case and control groups or between Ad41-positive patients and healthy controls. In addition to HAdV 40 and 41, HAdV 3 was also associated with diarrhea (OR = 17.301, adjusted OR = 9.205, *p* < 0.001).

**Conclusions:**

Our results demonstrate a high diversity of HAdV present among diarrhea and healthy children and implicate that non-enteric HAdV3 may lead to diarrhea.

## Background

Human adenoviruses (HAdV) is a major causes associated with a variety of diseases including acute respiratory illness, acute gastroenteritis, conjunctiva, hemorrhagic cystitis, hepatitis, hemorrhagic colitis, pancreatitis, nephritis, or meningoencephalitis [1]. Human adenoviruses (HAdV)belong to the genus Mastadenovirus of family Adenoviridae and consist of 7 species (HAdV-A through HAdV-G) including over 70 serotypes [2]. While different genotypes may cause different clinical features. HAdV F that includes serotypes 40 and 41, is connected with gastroenteritis, and thus is called an enteric adenovirus [3–5]. As previous study indicated, enteric adenovirus accounted for 1 to 20% diarrhea cases [6] and other serotypes were also identified sporadically in diarrhea patients, such as HAdV-A (12,18,31) HAdV-B (3,7,11,14,21,16) HAdV-C (1,2,5,6) and HAdV-D (8,9,10,28,29,30,32,37,43,46,61,64,70) and HAdV-G (52) [7–14], however, their role in causing diarrhea remains unclear due to most previous studies focused solely on diarrhea-causing pathogens while healthy controls were not recruited [10].

In this study, we conducted a preliminary matched case-control study to estimate the role of adenovirus and investigate the distribution and pathogenicity of adenovirus serotypes in diarrhea patients.

## Methods

### Study population and fecal specimens

A total of 634 fecal specimens were enrolled in this study, including 273 fecal samples collected from infants and children with acute gastroenteritis and 361 fecal specimens from the inpatient children in the surgical department and had no symptoms of diarrhea about half month before enrollment. All the samples were collected from Children’s hospital of Hebei Province (China) between June and November 2017. The study was conducted with the approval of the Ethics Committee of Children’s hospital of Hebei Province, and written informed consents were obtained from the children’s parents after informed them the use of data for analysis and using the results for improving patient care activities and without disclosing their names or identity.

### Sample preparation and nucleic acid extraction

About 2 g fecal samples were suspended in 1 ml Hanks balanced salt solution(HBSS), and centrifuged at 8000 rpm for 10 min then liquid supernatant was collected. Viral nucleic acid was extracted from the supernatant using Master Pure Complete DNA and RNA purification kit (Epicenter Technologies, Madison, WI) according to the manufacturer’s instructions, the extracts was eluted in 30 μl of DNase and RNase-free water and stored at − 80 °C until use.

### Detection and quantification of HAdV

HAdV was detected by quantitative real-time PCR (qPCR) as described in a previous study [15] using Premix Ex Taq™ (Probe qPCR) (Takara, Dalian, China) in a CFX96TM real-time system (BIORAD, USA) according to the manufacturer’s instructions. The number of copies in qPCR was calculated from standard curves of serial dilutions of a recombinant plasmid carrying synthetic target insert. Standard curves were generated by plotting the log of the starting quantity of a recombinant plasmid against the cycle threshold (Ct) value obtained from the amplification of each dilution. The qPCR positive samples were then retested by nested PCR and sequencing for serotyping as described in a previous study [16] The nested PCR was conducted using Premix Taq™ (TaKaRa Taq™ Version 2.0 plus dye) (Takara, Dalian, China) in a PCR Amplifier (Thermo Electron Corp., Vantaa, Finland) according to the manufacturer’s instructions. The primers/probes used in this study are listed in Table 1.

**Table 1:** Primer/probe used in qPCR and nest PCR

	Primer/probe	Sequence (5′-3′)	Reference
qPCR	F-primer	GCCCCAGTGGTCTTACATGCACATC	[15]
	R-primer	GCCACGGTGGGGTTTCTAAACTT	
	probe	FAM-TGCACCAGACCCGGGCTCAGGTACTCCGA-TAMRA	
nested PCR	AdhexF1	TICTTTGAC ATICGIGGIGTICTIGA	[16]
	AdhexR1	CTGTCIACIGCCTGRTTC CACA	
	AdhexF2	GGYCCYAGYTTYAARCCCTAYTC	
	AdhexR2	GGTTCTGTCICCCAGAGARTCIAGCA	

### Nucleotide sequence and phylogenetic analysis

**Fig. 1:**
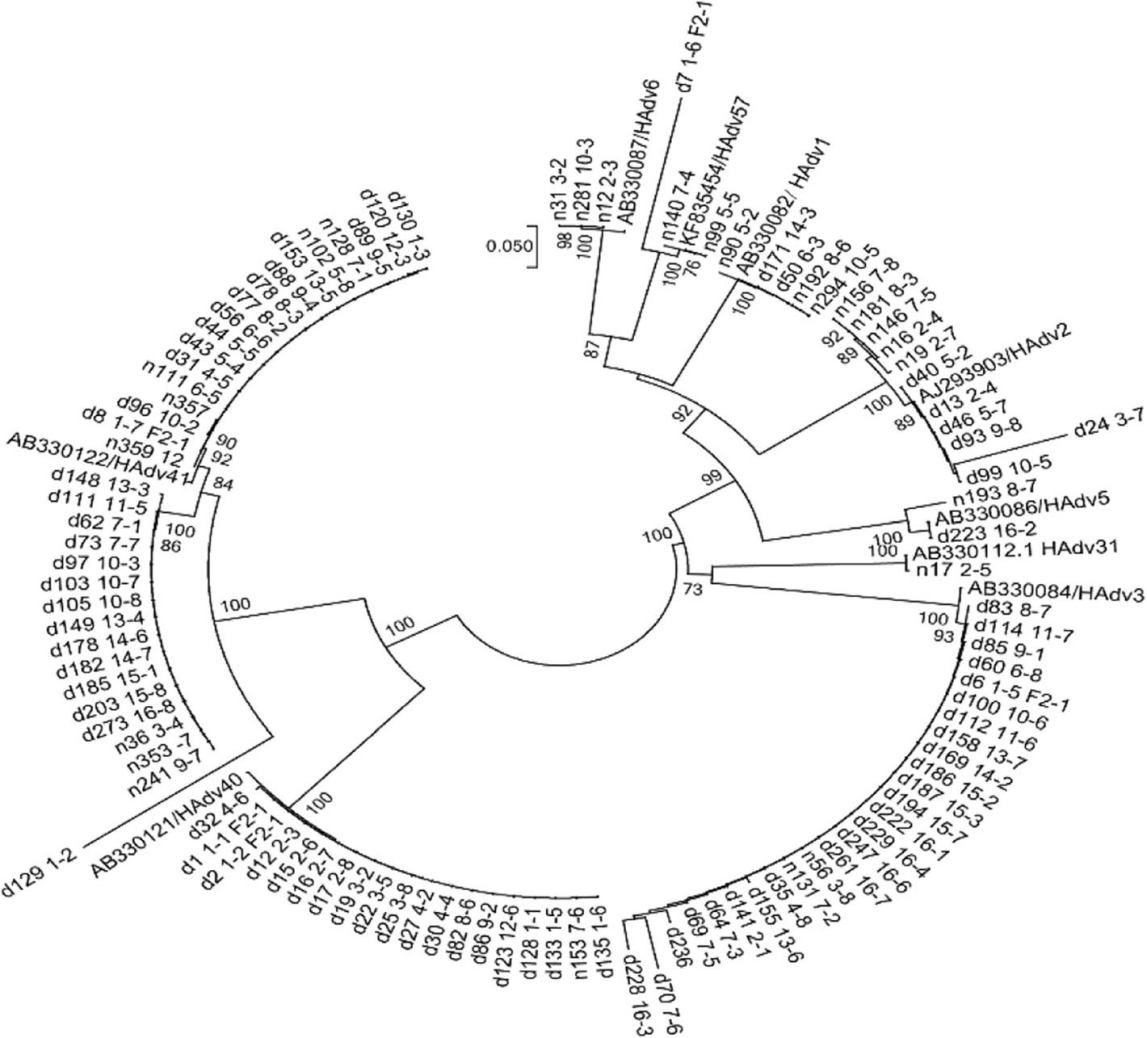
The phylogenetic tree of HAdV-positive samples

The nucleotide sequences of PCR products positive for HAdV were subjected to direct sequencing (TsingKe, Beijing, China) and analyzed by using the BLASTN program (available at: http://blast.ncbi.nlm.nih.gov/Blast). Sequence alignment was carried out in the BioEdit software version 7.2^29^. Phylogenetic and molecular evolutionary analyses were conducted using the MEGA (version 6.0), and Neighbor-joining (NJ) trees were constructed using the Kimura two- parameter method and the reliability was examined with bootstrap resampling 1000. The reference adenovirus strains and accession numbers used in this study were as follows; human adenovirus 1 (AB330082), human adenovirus 2 (AJ293903), human adenovirus 3 (AB330084), human adenovirus 5 (AB330086), human adenovirus 6 (AB330087), human adenovirus 31 (AB330112), human adenovirus 40 (AB330121), human adenovirus 41 (AB330122) and human adenovirus 57 (KF835454). The phylogenetic tree is shown in Fig. 1.

### Detection and quantification of other common diarrhea-associated pathogens

For those HAdV 3-positive samples, Rotavirus, norovirus GII, norovirus GI, sapovirus, astrovirus, adenovirus and Salmonella were detected using TaqMan probes for qPCR; Shigella, C. jejuni, and ETEC were detected using SYBR Green for qPCR according to the protocols previously published [17]. Human bocavirus was detected by using commercialized kits (Beijing Applied Biological Technologies Co.LTD, Beijing, China).

### Detection of mRNA of HAdV-3

The total RNA was extracted from 24 HAdV-3 positive samples by using QIAamp Viral RNA Mini Kit (designed for viral RNA extraction only). HAdV was detected by qRT-PCR using One Step Prime Script™ RT-PCR Kit (Takara, Dalian, China) in a CFX96TM real-time system (BIORAD, USA) [15]. To eliminate of impact of adenovirus DNA on the qRT-PCR results, we also detected DNA of these 24 HAdV-3 positive samples by using Premix Ex TaqTM (Probe qPCR) (Takara, Dalian, China) [15].

### Statistical analysis

Wilcoxon test was used to compare viral load and age between the two groups. The strength of association between diarrhea and potential risk factors of non-enteric adenoviruses was assessed by calculating an odds ratio (OR). Chi-square test was used to analyze sex distribution. Statistical significance was set at *p* < 0.05. The data analysis was conducted by SPSS software version 21.0.

## Results

### Prevalence of HAdV

HAdV were detected in 105(16.56%) among 634 clinical fecal specimens including 79(28.94%, 79/273) of these in diarrhea children and 26(7.20%, 26/361) in healthy children. Median age of patients positive for HAdVs was 8 months (range 5 months − 15 months) and 72 patients (91.14%, 72/79) were under 3 years while median age of healthy controls positive for HAdV was 18.5 month (range 10 months − 36 months) and 17 (65.38%, 17/26) were under 3 years. Of 79 positives for HAdV in diarrhea group, 58.28% (46/79) were male and 41.77% (33/79) were female (χ^2^ = 0.6373, *p* = 0.4247) while 57.69% (15/26) were male, 42.31% (11/26) were female (χ^2^ = 1.7583, *p* = 0.1848) in 26 healthy children positive for HAdV. Age and gender distribution of adenovirus positive did not show any statistically significant differences between the two groups as shown in Table 2.

**Table 2:** Characteristic of diarrhea children and healthy controls with HAdV positive

Characteristic	cases (*n* = 273)	controls (*n* = 361)
Age group		
≤ 3 years old	26.37%(72/273)	4.71%(17/361)
>3 years old	2.56%(7/273)	2.49%(9/361)
Gender		
Male	16.85%(46/273)	4.16%(15/361)
Female	12.09%(33/273)	3.05%(11/361)

### Molecular epidemiology

A phylogenetic tree according to partial hexon gene was constructed to identify HAdV species and serotypes. A total of 105 HAdV nucleotide sequences (820 bp) were compared to four different HAdV prototype strains (A, B, C, and F). Of 105 HAdV positive samples,9 different serotypes (HAdV 40, 41, 3, 2,1,5,57,6 and 31) were identified in control group, with Adv41 being the most frequently found. In diarrhea group, 7 different serotypes (HAdV 40, 41, 3, 2,1,5 and 57), with HAdV 40, 41and 3 being the most dominant, were identified. However, HAdV 6 and 31 occurred only in control group. The distribution of different HAdV serotypes between the two groups are summarized in Table 3 and Fig. 1.

**Table 3:** HAdV serotypes distribution between diarrhea group and healthy control group

HAdV serotypes	Diarrhea 28.94% (79/273)	Healthy control 7.20% (26/361)
Ad41	9.89%(27/273)	2.22%(8/361)
Ad40	6.59%(18/273)	0.28%(1/361)
Ad3	8.79%(24/273)	0.55%(2/361)
Ad2	2.20%(6/273)	1.39%(5/361)
Ad1	0.73%(2/273)	0.83%(3/361)
Ad5	0.37%(1/273)	0.28%(1/361)
Ad6	–	0.83%(3/361)
Ad57	0.37%(1/273)	0.55%(2/361)
Ad31	–	0.28%(1/361)

### Viral load of HAdV

The results of quantification of viral-load in 105 HAdV-positive were not provided. The result indicated no significance difference(z = − 1.611, *p* = 0.107) in the viral load was found between diarrhea group (79 positives) and control group (26 positives). We further assessed the viral load of HAdV 41, and also showed that there was no statistic difference between the two groups (Z = − 0.157, *p* = 0.875). The viral loads of other serotypes were not statistically analyzed, because of the small positive numbers of these serotypes.

### The result of mRNA of HAdV-3

The results indicated that 22 specimens were detected HAdV positive with Ct value less than 30, meaning the presence of adenovirus mRNA, it may suggest that the virus is replicated in the body. However, the result indicated that all the samples had Ct value greater than 36 with the DNA of the adenovirus were detected in the total RNA extracted, suggesting these samples were without or had a tiny amount of DNA of adenovirus.

### Risk assessment

We evaluated the risk factor of diarrhea among different serotypes of the HAdV using odds ratio (OR) calculation between the two groups. As shown in Table 4, HAdV serotypes 41, 40 and 3 were the high-risk factors for diarrhea apparently, while HAdV serotypes 1, 2, 5 and 57 seemed no association with diarrhea. And the OR values for serotypes 31 and 6 were not determined due to the detection failure in patients with diarrhea.

**Table 4:** The results of risk factors assessment among different HAdV serotypes

HAdV serotypes	*OR* value	95% CI	*p* value
41	4.843	2.164–10.839	< 0.001
40	25.412	3.371–191.572	0.002
3^a^	17.301	4.052–73.869	< 0.001
2	1.6	0.483–5.298	0.442
57	0.66	0.060–7.315	0.735
1	0.881	0.146–5.307	0.89
5	1.324	0.082–21.255	0.843

*CI* (Confidence interval)

a: the adjusted OR = 9.205 (CI 2.705–40.841) *p* < 0.001, excluding 10 samples co-infected with either of the other common diarrhea-associated pathogens as described in Section “Detection and quantification of other common diarrhea-associated pathogens”

### Other common diarrhea-associated pathogens in HAdV 3-positive samples

Among 24 HAdV 3-positive samples, other common diarrhea-associated pathogens (Rotavirus, norovirus GII, norovirus GI, sapovirus, astrovirus, bocavirus, Salmonella, Shigella, C. jejuni, and ETEC) were screened and 10 samples were found to be co-infected with either of Rotavirus (5), norovirus GII (3), C. jejuni (1), or Rotavirus and norovirus GII (1).

## Discussion

Despite the remarkable improvement in children’s public health worldwide, diarrhea is still the second most prevalent cause of death in children younger than 5 years [18]. Enteric viruses, especially rotavirus, have been considered as the leading pathogens of pediatric diarrhea worldwide [19, 20]. Other major enteric viruses causing childhood diarrhea included norovirus, human adenovirus(HAdV), human astrovirus, and sapovirus [17, 21–23]. To better understand the pathogens causing diarrhea is critical to prevent and control diarrhea disease [24].

Adenovirus serotypes 40 and 41 were considered to be the most remarkable pathogens associated with acute gastroenteritis [13]. A few studies reported the existence of non-enteric adenovirus in patients with diarrhea, but the role of non-enteric adenovirus played in diarrhea is unclear due to the lack of healthy control [6, 8–10, 12], A recent report conducted in Bangladesh, described the diversity of adenovirus pathogens and the age, gender, major clinical symptoms of adenovirus positive diarrhea children, but lack of matched controls to determine the relationship between adenovirus and diarrhea, this study may not provide a better speculation of adenovirus associated diarrhea [10]. A previous study reported that the positive rate of adenovirus were significantly different between community-acquired diarrhea and hospital-acquired diarrhea among children in Beijing china [8], and the adenovirus serotypes identified appeared to be similar to our study. To our best knowledge, this is the first matched case-control study on the aetiology of non-enteric adenovirus in children in china. The aim of this study is to exhibit the diversity of adenovirus in diarrhea children and healthy children then reveal the relationship between non-enteric adenovirus and diarrhea.

A total of 7 adenovirus serotypes including 5 non-enteric adenovirus types were found in diarrhea children. Previous epidemiologic studies demonstrated HAdV (40/41) were dominant in adenovirus-associated diarrhea [25–27]^,^ our survey of adenovirus serotypes in diarrhea children indicated HAdV (40/41) accounted for 56.96% (45/79), which coincides with previous reports. Interestingly, adenovirus serotype 3 (30.38%, 24/79) unexpectedly exceeded serotype 40 (22.78%, 18/79) and became the second leading subtype in diarrhea children. Additionally, HAdV serotypes 1,2 and 5 were detected in diarrhea children, which is consistent with published studies [10] while adenovirus serotype 57 was first reported. In the case of healthy children group, HAdV 41 (30.77%, 8/361) had the highest prevalence. In addition, HAdV serotypes 1, 2, 3, 5, 6, 31, 40 and 57 were also appeared in the healthy population, particularly, HAdV 6 and 31 were present uniquely in control group in this study. This added up to a total of 9 serotypes including 2 enteric adenovirus types and 7 non-enteric adenovirus serotypes in control group. These adenovirus serotypes are more likely to represent a portion of the normal human virome in the gut, owing to their ability to establish persistent infections. We also compared age and gender distribution of adenovirus infection among diarrhea children and healthy children. Our results suggested adenovirus infection prevalence is higher among younger children (under 3 years) than that of the relatively older children (over 3 years), the prevalence of enteric adenoviruses was identified to be significant higher in diarrhea children (28.94%) than in healthy children (7.20%) and no statistically significant difference in gender distribution of adenoviruses infection was observed in both groups. These results were in accordance with previous studies [9, 28, 29].

The viral load may vary at different stages of the disease and may be associated with clinical symptoms. Apparently, the qPCR assay has already become the gold standard for viral load quantification based on a standard curve [30]. In order to estimate the role of viral load of adenovirus between diarrhea group and healthy group, we quantified the adenovirus-positive samples by qPCR and analyzed the data by Wilcoxon test, our results indicated that there was no statistical difference in the viral load between the two groups, implying viral load itself was not a major factor leading to dominant infection or recessive infection. To be further, due to relatively small positive number of other adenovirus serotype (except serotype 41) infection in both groups, we just compared the viral load of adenovirus serotype 41 between the diarrhea group and healthy children group and revealed no difference between the two groups. This result suggested viral load of adenovirus 41 alone appeared not necessarily associated with acute gastroenteritis and adenoviruses 41 also seems resident of the human gut. The pathogenicity of enteric adenoviruses (such as HAdV41) might be attributed to the difference in the genome-wide composition or the susceptibility among the diarrhea and healthy population.

To further investigate the role of adenovirus in the diarrhea, we performed the comparison of the risk assessment on the same serotype pathogens of adenovirus present in the two groups by calculating the OR value. The subtype 40 and 41 of adenovirus were significantly correlated with diarrhea as expected. Surprisingly, non-enteric adenovirus subtype such as HAdV 3 was demonstrated to be a high-risk factor for diarrhea (*OR* = 17.301). To exclude the effect of co-infection with HAdV 3 on the diarrhea, 24 HAdV 3-positive samples were retrospectively tested for other common diarrhea-associated pathogens,10 samples with co-infections were not counted. The adjusted *OR* = 9.205 was thus obtained based on the remaining 14 samples. Unlike most prior studies that HAdV 3 was a common pathogen causing respiratory disease [31, 32] and seemed no relationship with diarrhea [10, 11], our results suggest a strong association of HAdV 3 with diarrhea. Other serotypes (HAdV 1,2,5,57) of non-enteric adenovirus were less or equally detected in both groups, indicating no link with diarrhea.

There were a few limitations in our study. First, all the patients presented diarrhea came from outpatient department of the same region without detailed information on the clinical symptoms, treatment and recovery. Second, we just collected fecal specimens during a 6-months period, larger sample size is needed in the future to warrant more benefit from this study. Third, we did not detect enteric viruses especially the other common pathogens associated with pediatric diarrhea for all the samples, thus the role of co-infection with adenovirus remains unknown.

## Conclusions

In conclusion, our study demonstrates age and gender distribution, viral load and molecular epidemiology of adenovirus infections among diarrhea patients and healthy children in developing country and provides supplements of adenovirus infection in healthy control to study the relationships between adenovirus and diarrhea. Our results elaborate a high diversity of HAdVs present among diarrhea and healthy children and implicate that non-enteric HAdV 3 may lead to diarrhea.

## Abbreviations

CI: Confidence interval
ETEC: Enterotoxigenic *E.coli*
HAdV: Human adenovirus
OR: Odd ratio
PCR: Polymerase Chain Reaction
qPCR: quantitative real-time PCR
